# Population profiling in China by gender and age: implication for HIV incidences

**DOI:** 10.1186/1471-2458-9-S1-S9

**Published:** 2009-11-18

**Authors:** Yuanyi Pan, Jianhong Wu

**Affiliations:** 1 Centre for Disease Modeling, Department of Mathematics and Statistics, York University,Toronto, Ontario, Canada

## Abstract

**Background:**

With the world's largest population, HIV spread in China has been closely watched and widely studied by its government and the international community. One important factor that might contribute to the epidemic is China's numerous surplus of men, due to its imbalanced sex ratio in newborns. However, the sex ratio in the human population is often assumed to be 1:1 in most studies of sexually transmitted diseases (STDs). Here, a mathematical model is proposed to estimate the population size in each gender and within different stages of reproduction and sexual activities. This population profiling by age and gender will assist in more precise prediction of HIV incidences.

**Method:**

The total population is divided into 6 subgroups by gender and age. A deterministic compartmental model is developed to describe birth, death, age and the interactions among different subgroups, with a focus on the preference for newborn boys and its impact for the sex ratios. Data from 2003 to 2007 is used to estimate model parameters, and simulations predict short-term and long-term population profiles.

**Results:**

The population of China will go to a descending track around 2030. Despite the possible underestimated number of newborns in the last couple of years, model-based simulations show that there will be about 28 million male individuals in 2055 without female partners during their sexually active stages.

**Conclusion:**

The birth rate in China must be increased to keep the population viable. But increasing the birth rate without balancing the sex ratio in newborns is problematic, as this will generate a large number of surplus males. Besides other social, economic and psychological issues, the impact of this surplus of males on STD incidences, including HIV infections, must be dealt with as early as possible.

## Background

HIV/AIDS spread in China has been an increasingly growing concern since the first AIDS case was reported in Beijing [1]. When a joint assessment by the Chinese government, UN and WHO suggested in 2005 [2] that there were 650,000 individuals living with HIV/AIDS and 75,000 had developed AIDS, some claim that future incidence could rise up to 10-15 million cases by 2010 [3]. With the largest population in the world, China must maintain and strengthen its efforts to control the epidemic including raising awareness and prevention of the disease among risk groups: injecting drug users (IDUs), former plasma donors, men who have sex with men (MSM), sex workers, etc. Another possible high-risk group that is rarely studied is the surplus men. According to a study by J.D. Tucker and colleagues in 2005 [4], most people in this group are young, unmarried, poor, unemployed and less educated and are therefore more likely to turn to sex workers; consequently, the number of sex workers is likely to be increased due to the sexual demand from surplus men. Both factors increase the average sexual partner exchange rate, and a higher partner exchange rate normally leads to a larger HIV incidence rate [5].

However, sex ratio in the human population is often assumed to be 1:1 in most studies of sexually transmitted diseases (STDs). More specifically, the population is generally divided into certain subgroups based on gender, sexual behaviour and stage of disease development; the change rate of population in each subgroup is often assumed to be a function of the natural population growth, the nature of disease and the size of all other groups; the natural growth of the human population is usually believed to be proportional to the size of total population [5-9] (and references therein); and the sex ratio is balanced. This is normally true for a society with a stable age structure. But it is different for countries with rapid social and economical changes. For example, according to United Nation [10], the male population in China in the sexually active stage will decrease from 29% in 2000 to 23% in 2030, while the sex ratio in the same group will increase from 1.066 to 1.12.

There are many problems that might be caused by gender imbalance, such as antisocial behaviour and violence threatening the social stability, women trafficking and expansion of the sex industry [11]. Another major issue that deserves serious attention is the impact of gender imbalance on the spread of STDs, especially those STDs such as HIV/AIDS with long incubation periods and with different stages of disease progression.

Although a study proposed by T. E. Senn *et al*. [12] suggests that there is no association between the sex ratio and the number of sexual partners in African Americans, it is commonly believed that the surplus men will turn to sex workers for sexual service, especially when they cannot afford regular sex partners [4]. A. Ebenstein and E. Jennings in 2008 [13] found that most counties with a high rate of single men tend to be regions with a high rate of men paying for sex. They also claimed that 9% of men will pay for sex in 30 years, rising from 6% in 2005, partially because of the increasing number of unmarried men. Other reasons for the ascending trend of commercial sex activity include various social and cultural changes driven by economic reform since 1976. The high rate of men purchasing sex seems to be occupation related. In [13], the authors noted the relationship between the percentage of men working in the construction industry and the prevalence of commercial sex activity. Other relevant studies include the work of B. Wang *et al*. [14] and a report by R. Zhao *et al*. [15]. The former found 10% of a total of 1,304 rural-to-urban migrant men paid for sex; the latter claimed that 9.4% of 232 miners in townships of Yunnan province purchased sex, while 9% of Chinese men in a similar age range purchased sexual services [16]. Regardless of the different focuses of these studies, all authors seem to agree that male clients of female sex worker in China need to be studied more, given the high rate of HIV/AIDS infection and its role of bridging the high-risk groups and the general population in the HIV/AIDS transmission. When surplus men become surplus clients [17], HIV/AIDS incidences may rise rapidly.

The disproportionate surplus men may also increase homosexual activities. Although there is no solid evidence as far as we know that suggests surplus men in China have higher level of homosexual behaviours than other demographic groups, especially when the sex service is sufficiently supplied by female sex workers, it is wildly believed that homosexual behaviours are common in certain male concentrated places like prison [18] and the army [19]. Nevertheless, the general public may become more tolerant to homosexual behaviour and more hidden homosexuals may publicly express their sexualities [11]. Since unprotected same-sex behaviour is well known for its high risk in spreading HIV/AIDS [20], an extraordinarily off-balanced male population may contribute to the increase of HIV/AIDS in the future. It is therefore important to make accurate predictions of the profile of populations in terms of gender and stages of sexual activities. This is certainly a very difficult task. For example, the UN claimed in 1951 that the world population in 30 years would be 2.976 - 3.636 × 10^9 ^[21] but it was 4.45 × 10^9 ^in 1980. We note that the 1986 UN estimation of the world population 6 × 10^9 ^in 2000 [22] was close to the actual number, 6.1 × 10^9^. Predicting the population in countries such as China is certainly no less challenging, though some efforts have been made [23-26].

**Figure 1:**
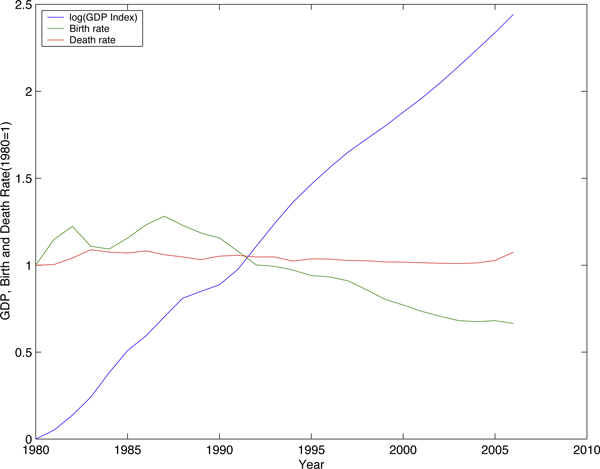
**GDP index, birth rate and death rate in China. Source: Statistics China **[27].

One factor contributing to the difficulty in predicting the population is the fast-growing economy. In less than 30 years, China has increased its Gross Domestic Product (GDP) from 364.52 billion yuan in 1976 to 21,087.1 billion yuan in 2006. GDP index per capita in 2006 is almost 10 times that of 1976 [27]. The skyrocketing economy brought Chinese people a better life, as well as a lower birth rate that has been observed in developed countries during their development (Figure 1). The negative correlation between economic success and population growth is probably due to the trade-off between quantity and quality of children [28,29]. It would be difficult to use this correlation alone to predict the population in China, since not only the quality of raising children but also the rate of consumption of other commodities is expected to be different in coming decades.

Another reason that we should not consider the number of children just as a function of economy is the China's unique one-child policy. In 1970s, China's leaders worried that its swelling population could consume any effect to improve the quality of Chinese people's life. This worry was based on Malthusian Theory [30], which argues that population grows geometrically and that the food supply increases only at an arithmetic rate. Famine is hence inevitable unless constraints are applied to population growth. A planned-birth policy was introduced in 1973 and the one-child-per-couple policy became a national law in 1980 [31]. Although certain exceptions exist among ethnic minorities and in rural areas [32], most couples in China only have one child. Adding to the difficulty of predicting the population size in China is the abnormal gender imbalance among newborns. It is reported, for example, by Statistics China, that the average male-female ratio was 121.8 from 2003 to 2007 [27].

**Figure 2:**
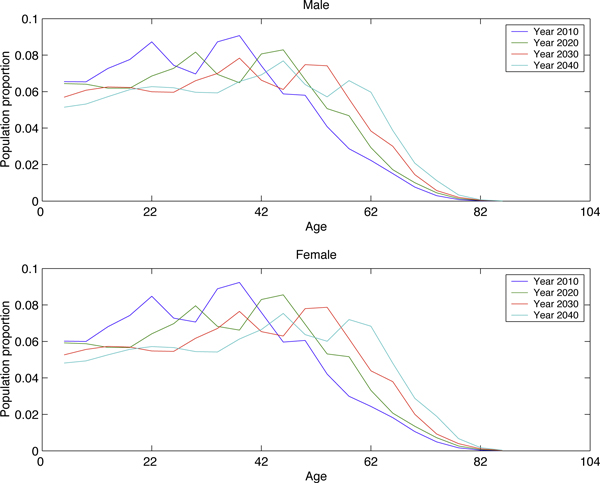
**Age structure of male and female in China. Source: UN **[10].

In this study, we compartmentalize the total population into 6 sex-age subgroups. The age structure is explicitly introduced because of a possible unstable age structure in the coming decades illustrated in Figure 2. A possible reason for the changing age structures is the comparatively large variance in the birth rate back in 1980s and 1990s, as shown in Figure 1. Our model incorporates the sex-ratio imbalance in newborns. Although this ratio is normally around 1.05 in a large population [33], China has a much higher sex ratio: 1.15 estimated by the UN [10]; data from Statistics China is even more dramatic [27]. A similar phenomenon seems to have been reported in India (1.08), Mauritania (1.08) and South Korea (1.1). Fertility decline and son preference are wildly believed to be two main reasons [34] to select sex in birth. Li *et al*. [35] established some quantitative relations among sex ratio at birth, fertility, and son preference. China, as one can imagine, has the worst scenario due to the one-child-policy and its long-lasting son preference [36].

Existing studies of the population size with consideration of gender imbalance include the work of Yellin *et al*. [37], where a dynamic system with three compartments - single individuals with two sexes, *N*_1 _and *N*_2_, and married couples, *T*_3 _- is constructed. A match function with variables *N*_1 _and *N*_2 _was introduced in order to estimate the new cases in *T*_3_. Our study differentiates from the aforementioned work by considering the rate of newborns as a function of the sizes of sexually active male and female populations. This is described by our proposed matching function, that needs to incorporate the so-called minimum viable population (MVP) in order to avoid the singularity at the trivial equilibrium state. Such an MVP size was introduced in [38-40] to represent the minimum number of individuals in a population in order for the species under consideration to survive.

## Methods

### Mathematical model

The total population is divided into 6 subgroups determined by gender (Male (M) and Female (F)) and stage of ages (stage 1: age 0 to age 14; stage 2: age 15 to age 49; and stage 3: age 50 and beyond). The second group will be important for reproduction and sexually transmitted infections. The sizes of these subgroups will be denoted by *M*_1_, *M*_2_, *M*_3_, *F*_1_, *F*_2_, *F*_3_, respectively. For example, *M*_2 _is the size of the male population aged 15 to 49.

The population change of each subgroup is the number of individuals entering minus those leaving this subgroup. More specifically, the inflow to stage 1 is due to the birth of newborns, while the inflow to and the outflow from stages 2 and 3 are due to aging and death. Note that people only leave age group 3 due to death.

The number of newborns is determined by a matching function *C *:= *C*(*M*_2_, *F*_2_), similar to the match function introduced in [37], of the populations in stage 2 in both genders. It is natural to require the following properties of the matching function:

• *C*(*M*_2_, *F*_2_) = 0 if either *M*_2 _= 0, or *F*_2 _= 0, or both;

• *C*(*M*_2_, *F*_2_) is an increasing function of either variable if another is fixed;

• *C *∝ *M*_2 _if *F*_2 _≫ *M*_2_, and *C *∝ *F*_2 _when *M*_2 _≫ *F*_2_.

Two obvious functions satisfying the above properties are *C *∝ min(*μ*_*M*_*M*_2_, *μ*_*F*_*F*_2_) and  where *μ*_*M *_and *μ*_*F *_are the average number of reproductive partners that one individual can have. In our simulations, we choose the second one due to its smoothness. In this case, since monogamy is still the main marriage system in the modern society, we assume that *μ*_*M *_= *μ*_*F *_= 1.

The above function has a singularity at the equilibrium when *M*_2 _= *F*_2 _= 0. On the other hand, as stage 2 in our model is responsible for reproduction, it is natural to assume a minimal size must be maintained at this stage. Following the theory of minimum viable population (MVP) (see [38-40]), we will use the modified function , where ϵ is a small positive number: the specific value of ϵ is not important for our simulations and analysis, as long as it is relatively small. This implies that *C*(*M*_2_, *F*_2_) ≈ 0 if *M*_2 _+ *F*_2 _<< ϵ. Furthermore, the population growth is limited by the carrying capacity [41] so that the reproduction rate decreases when the population size is greater than the capacity. Consequently, we will use the following matching function throughout the reminder of this paper:

with *N *being the size of the total population and *K *relevant to the carrying capacity.

Therefore, the deterministic compartmental model for a population stratified by genders and ages is as follows:

### Parameters and their estimation

A description of the model parameters, along with their values (to be used in our simulations) and relevant references, can be found in Table 1. Note that  is the number of newborns per unit time. In our simulations, we estimate the value of *μ *and *K *by using the nonlinear least square estimation (LSE) to estimate the model-generated numbers against the reported newborns in 2003, 2004 and 2005 (the initial value of *K *comes from [42], and the initial value of *μ *is determined by taking the initial *K *and other parameters in 2003). Note that, unless otherwise specified, values of other parameters are taken from [27].

**Table 1:** Parameters estimated from Statistics China [27].

Parameter	Description	Value	Unit	Estimation description
*μ*	Birth rate	0.11584	Proportion	Estimated using LSE
*σ*	Males in newborns	0.5487	Proportion	Average ratio of age 0-4 in 2003-2007
	Growth rate of age group 1	1/15	Proportion	Inverse of time in age group 1
	Growth rate of age group 2	1/35	Proportion	Inverse of time in age group 2
	Death rate of male in age group 1	0.00122	Proportion	Average rate in 2003-2007
	Death rate of female in age group 1	0.00122	Proportion	Average rate in 2003-2007
	Death rate of male in age group 2	0.0022	Proportion	Average rate in 2003-2007
	Death rate of female in age group 2	0.0012	Proportion	Average rate in 2003-2007
	Death rate of male in age group 3	0.0242	Proportion	Average rate in 2003-2007
	Death rate of female in age group 3	0.0189	Proportion	Average rate in 2003-2007
ϵ	MVP	0.05	10^4 ^individuals	Source: [48]
*K*	Carrying capacity parameter	549186	10^4 ^individuals	Estimated using LSE
*M*_1_	Males in age group 1	14072.82	10^4 ^individuals	Initial population in 2003
*F*_1_	Females in age group 1	12211.95	10^4 ^individuals	Initial population in 2003
*M*_2_	Males in age group 2	37333.68	10^4 ^individuals	Initial population in 2003
*F*_2_	Females in age group 2	36286.94	10^4 ^individuals	Initial population in 2003
*M*_3_	Males in age group 3	14563.88	10^4 ^individuals	Initial population in 2003
*F*_3_	Females in age group 3	14744.8	10^4 ^individuals	Initial population in 2003

## Results and Discussion

**Figure 3:**
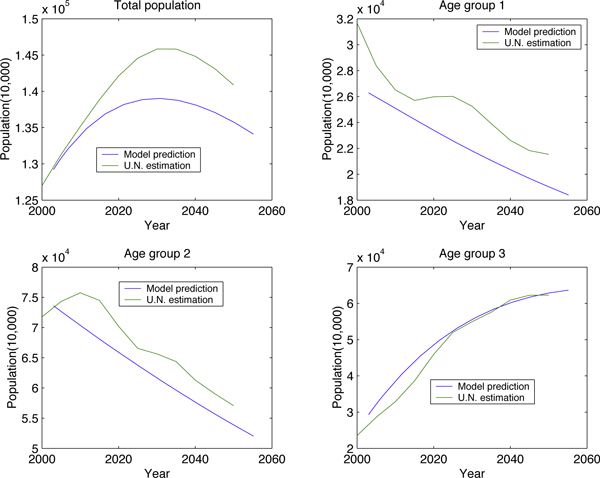
**Model estimation v.s. U.N. by population density in each age groups**.

**Figure 4:**
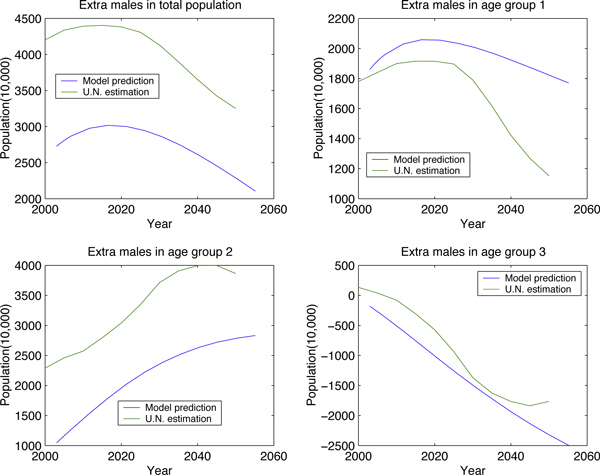
**Model estimation v.s. U.N. by number of extra males in each age group**.

Simulations based on our models show that China's population will decrease around 2030, which is consistent with the estimation from UN; see Figure 3. Other agreements include the changing trend of each age group and gender group, see Figure 4.

**Figure 5:**
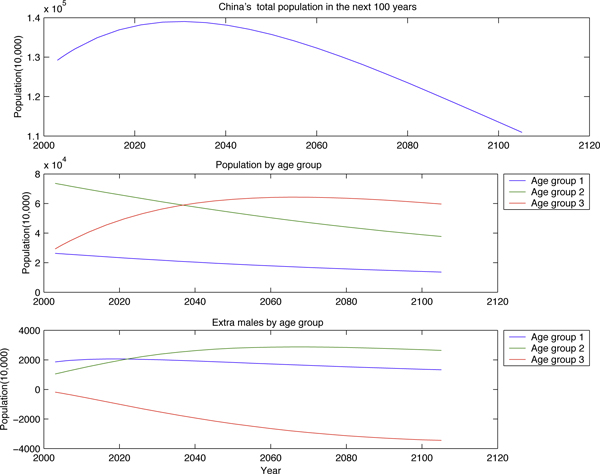
**China's population in 100 years**.

Assuming that the birth ratio and birth rate remain unchanged for the next 100 years, we examine how the China's population and its age-sex structure change in the future. We can see from Figure 5 that China's population will decrease around the year 2030. We can also see an aging China coming: a China with a shrinking population and with a larger portion of aged individuals.

The main motivation for this study is the sex imbalance; we will address the impact of this imbalance on the spread of STDs in a subsequent study. Our simulations show that there will be about 28 million male individuals in 2055 who will not have female partners during their sexually active ages (Figure 4). In China, these male individuals are called Guanggun, that is, bare branch. The social, economic and health issues caused by this subpopulation must be addressed.

**Figure 6:**
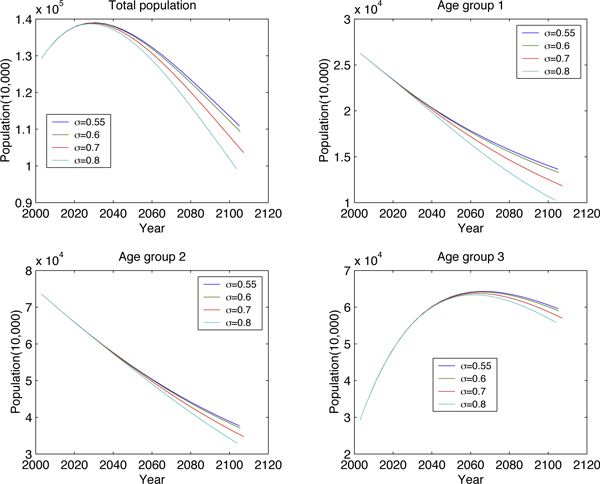
**Long-term effect to age group by varying *σ***.

**Figure 7:**
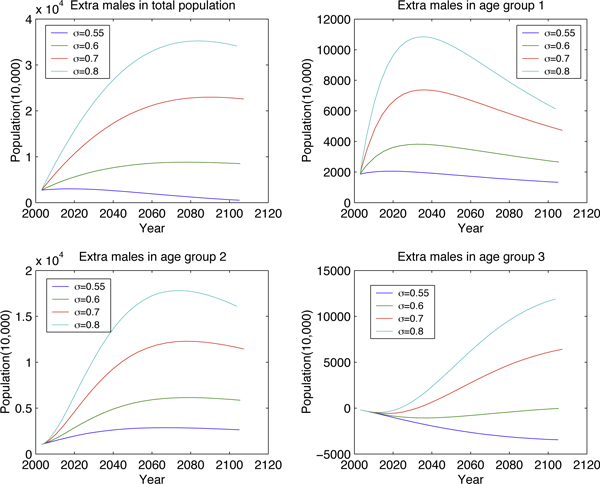
**Long-term effect to the number of extra males by varying *σ***.

**Figure 8:**
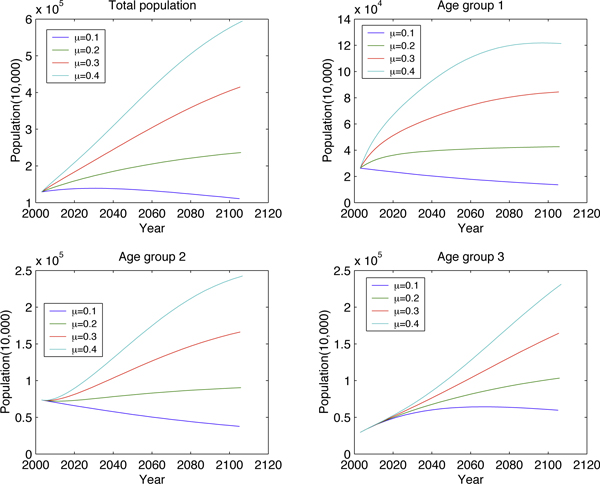
**Long-term effect to age group by varying *μ***.

**Figure 9:**
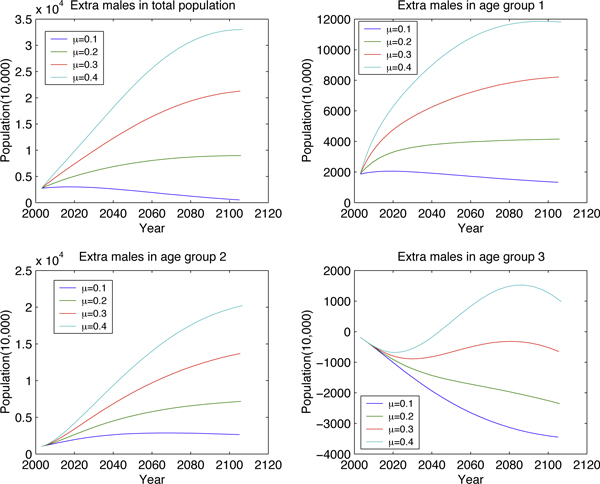
**Long-term effect to the number of extra males by varying *μ***.

Fixing *μ *(birth rate) and varying *σ *(proportion of newborn males) (Figure 6 and Figure 7) illustrates how the high sex-ratio imbalance in newborns affects the population structure. Similar manipulations to *μ *are presented in Figure 8 and Figure 9. These figures show that *σ *is less significant than *μ *in changing population size but it will tremendously deteriorate the sex ratio in adults.

## Conclusion

Unlike other countries, China has a tough and much-criticized policy to control its population growth. It is the national law that most couple in China can have only one child. This policy was effectively executed: total fertility has dropped from 4.86 in 1970s to 1.70 in the 21st century [10]. Meanwhile, the world's total fertility is 2.65 and that of developed countries is 1.56. Not intending to address all the controversies around this policy, we attempt to describe the population profiles under this policy and the related sex-ratio imbalance in newborns.

We found that this sex-ratio imbalance will decrease the population growth when the birth rate is fixed, although this negative effort can be balanced by increasing the birth rate. There will be an abnormally large number of male individuals without female partners during their sexually active stages in the next several decades if the sex-ratio imbalance in newborns is not reduced. The sexual demands from these surplus men may increase the high-risk heterosexual or homosexual activities and thereafter increase HIV/AIDS and other STD/STI incidences. The impact of these surplus males on STD incidence should therefore be addressed urgently.

There are also other interesting sociological and psychological issues arising from the shortage of women. Discrimination against women may be undermined [43]; bride trading was reported [44]; polyandry may be normalized [45]; crimes against women such as rape may rise [46], although some researchers claim that rape against women may decline because of the booming sex industry [47]. More efforts are required to examine each of these issues.

## Competing interests

The authors declare that they have no competing interests.

## Authors' contributions

Both authors contributed to the formulation of mathematical models. YP designed and conducted the model-based simulations and the estimation of model parameters from the literature.
